# Can coenzyme Q10 alleviate the toxic effect of fenofibrate on skeletal muscle?

**DOI:** 10.1007/s00418-023-02205-5

**Published:** 2023-06-04

**Authors:** Dalia R. El-Bassouny, Alyaa A. Mansour, Amany S. Ellakkany, Nasra N. Ayuob, Amany A. AbdElfattah

**Affiliations:** 1 Medical Histology & Cell Biology Department, Faculty of Medicine, Mansoura University, Mansoura, Egypt; 2 Medical Histology Department, Faculty of Medicine, Damietta University, Damietta, Egypt; 3 Department of Basic Medical Sciences, Faculty of Medicine, King Salman International University, South Sinai, El-Tor, Egypt; 4 Yousef Abdullatif Jameel Chair of Prophetic Medical Applications (YAJCPMA), Faculty of Medicine, King Abdulaziz University, Jeddah, Saudi Arabia

**Keywords:** Skeletal muscle, Fenofibrate, CoQ10, Caspase-3, Creatine kinase, Ultrastructure

## Abstract

Fenofibrate (FEN) is an antilipidemic drug that increases the activity of the lipoprotein lipase enzyme, thus enhancing lipolysis; however, it may cause myopathy and rhabdomyolysis in humans. Coenzyme Q10 (CoQ10) is an endogenously synthesized compound that is found in most living cells and plays an important role in cellular metabolism. It acts as the electron carrier in the mitochondrial respiratory chain. This study aimed to elucidate FEN-induced skeletal muscle changes in rats and to evaluate CoQ10 efficacy in preventing or alleviating these changes. Forty adult male rats were divided equally into four groups: the negative control group that received saline, the positive control group that received CoQ10, the FEN-treated group that received FEN, and the FEN + CoQ10 group that received both FEN followed by CoQ10 daily for 4 weeks. Animals were sacrificed and blood samples were collected to assess creatine kinase (CK). Soleus muscle samples were taken and processed for light and electron microscopic studies. This study showed that FEN increased CK levels and induced inflammatory cellular infiltration and disorganization of muscular architecture with lost striations. FEN increased the percentage of degenerated collagen fibers and immune expression of caspase-3. Ultrastructurally, FEN caused degeneration of myofibrils with distorted cell organelles. Treatment with CoQ10 could markedly ameliorate these FEN-induced structural changes and mostly regain the normal architecture of muscle fibers due to its antifibrotic and antiapoptotic effects. In conclusion, treatment with CoQ10 improved muscular structure by suppressing oxidative stress, attenuating inflammation, and inhibiting apoptosis.

## Introduction

Hyperlipidemia is the principal cause of atherosclerosis, which mostly leads to coronary heart disease. Changing the lifestyle is considered the first choice for the treatment of hyperlipidemia. However, in uncontrolled cases, lipid-lowering drugs help control elevated lipids levels. Statins mainly lower cholesterol levels, while fibrates mainly lower fatty acids and triglycerides (TG) (Tarantino et al. 2017; Kim et al. 2019).

Fenofibrate (FEN) is a synthetic fibric acid derivative that is transformed in the liver to its active form, fenofibric acid. It is a peroxisome proliferator-activated receptor-α (PPAR-α) agonist that activates genes responsible for fatty acid metabolism as mitochondrial β-oxidation (Sohn et al. 2017). FEN increases the activity of the lipoprotein lipase enzyme, thus enhancing lipolysis, reducing the level of TG-rich lipoproteins, and elevating the level of high-density lipoproteins (HDL) concentrations, implementing additional protective effects on the cardiovascular system (Emami et al. 2020). Despite all these beneficial effects, FEN may cause myopathy and rhabdomyolysis in rats and humans (Okada et al. 2009; Wang and Wang 2018).

Coenzyme Q10 (CoQ10) is an endogenously synthesized benzoquinone compound, widely known as ubiquinone, that is found in most living cells in the body. It is the only lipid-soluble antioxidant that animal cells synthesize de novo. It is primarily present in the mitochondria and acts as a cofactor in ATP generation via its pivotal role as an electron carrier in the mitochondrial respiratory chain (Garrido-Maraver et al. 2014). It is also found in the cell membrane and represents a principal molecule in the maintenance of the antioxidant system for protecting phospholipids of membranes from peroxidation, acting as a membrane stabilizer for the cell membrane and all intracellular membranes. In addition, it can protect membrane proteins and deoxyribonucleic acid (DNA) against oxidative damage (Silva et al. 2022).

The major form of CoQ10 found in the living organism is the reduced active form, ubiquinol (CoQH2), which is primarily responsible for its antioxidant properties (Onur et al. 2014). Therapeutic benefits of CoQ10 were demonstrated in aging-related disorders, mainly those accompanied by increased oxidative stress (Diaz-Casado et al. 2019) and in the mitigation of heavy metal toxicities from lead and arsenic due to its antiinflammatory, antioxidant, and antiapoptotic properties (Al-Megrin et al. 2020; Silva et al. 2022).

Therefore, this study aimed to elucidate skeletal muscle changes in adult male albino rats after FEN administration and to evaluate CoQ10 efficacy in preventing or alleviating these changes.

## Material and methods

### Chemicals

Fenofibrate (Lipanthyl®, 300 mg/capsule) was purchased from Minapharm, under the license of Laboratories Fournier, France. Coenzyme Q10 (30 mg/gelatin capsules) was purchased from Arab Company for Pharmaceuticals and Medicinal Plants.

### Animals and experimental design

Forty male rats (3–4-month-old, 180–200 g) were obtained from Mansoura Experimental Research Center (MERC). The rats were kept in an air-conditioned laboratory at 20–22 °C under 12 h light/dark cycles. A standard commercial pelleted diet and water ad libitum were used. Rats were allowed to acclimate for 2 weeks before the beginning of the experiment. The experimental protocol was approved by Mansoura Faculty of Medicine Institutional Research Board (IRB) (code number: MD/17.12.42). The experiment was performed according to the international guidelines for the use of laboratory animals.

After the acclimatization period, rats were divided, at random, into four groups: the negative control group (ten rats), receiving saline; the positive control (CoQ10) group (ten rats), receiving CoQ10 at a dosage of 100 mg/kg/day of CoQ10 powder dissolved in 2 ml 0.5% carboxymethyl cellulose (CMC) by gastric tube for 4 weeks (Haredy et al. 2017); the FEN group (ten rats), receiving FEN at a dosage of 60 mg/kg/day dissolved in 2 ml of 0.5% CMC once daily by gastric tube for 4 weeks (Pettersen et al. 2012); and the FEN + CoQ10 group (ten rats), receiving daily FEN at a dose similar to the FEN group followed by CoQ10 at a dose similar to the positive control group.

At the end of the experiment, the animals were anesthetized by intraperitoneal (IP) injection of sodium pentobarbital (40 mg/kg) (Kao et al. 2006).

### Biochemical assessment

Blood samples were collected from the tail vein of all animals to estimate the plasma level of creatine kinase (CK) as a biomarker for myopathy and muscle breakdown (Jansone et al. 2016) using a commercial kit (MAK116) from Sigma-Aldrich (St. Louis, MO, USA).

### Obtaining the samples

Rats were sacrificed and a part of the soleus muscle of the right hind limb of all animals were dissected and put in the cryostat for determination of succinic dehydrogenase enzyme (SDH) to differentiate between type I and type II fibers. Type 1 fibers are more susceptible to fibrate-induced muscle toxicity, so the soleus muscle was selected for this study as it is mainly formed of type 1 fibers (Okada et al. 2009). Other muscle specimens were put in 10% neutral buffer formalin (NBF) and processed to prepare paraffin sections for immunohistochemistry. The rats were then perfused through the left ventricle with 500 ml of phosphate buffer (0.1 mol) containing 2.5% glutaraldehyde and 2% paraformaldehyde (Monteiro et al. 2008). The soleus muscle of the left hind limb of all animals was obtained. Parts of the muscle were fixed in 10% NBF and processed to prepare paraffin sections for light microscopy. Other small muscle specimens (1 mm) were put in a mixture of 2.5% glutaraldehyde and 2% paraformaldehyde, postfixed in 1% osmium tetroxide, and processed to obtain semi-thin sections (toluidine blue stained) and ultrathin sections for electron microscopy.

### Light microscopy examination

Muscle specimens were fixed in 10% NBF for 24 h, dehydrated, cleared, and embedded to prepare a paraffin block that was sectioned giving 5 μm thick paraffin sections. The sections were stained with hematoxylin and eosin stain (H & E) for routine histological study (Bancroft and Layton 2019b) and Mallory’s trichrome stain, for demonstration of collagen fiber (Bancroft and Layton 2019a).

### Histochemical study

The muscle specimens were put in the cryostat (at −15 °C) and fresh cryocut sections, 10 µm thick, were cut and prepared for SDH histochemical study (Highley and Sullivan 2019).

### Immunohistochemical study

Another set of paraffin sections were immunohistochemically stained with the apoptosis marker caspase-3, using the peroxidase-labeled streptavidin–biotin technique (Sanderson et al. 2019). Sections were deparaffinized in xylol and rehydrated with alcohol. They were incubated with 3% H_2_O_2_ for 10 min for blockage of endogenous peroxidase activity. Slides were rinsed for 10 min in phosphate-buffered saline (PBS) at pH 7.4, then they were put in bovine serum albumin (1%) dissolved in PBS at 37 °C to minimize nonspecific background staining. Antigen retrieval was achieved by heating sections in citrate buffer (10 mmol/l). Afterward, monoclonal anti-caspase-3 antibody (at a dilution of 1:100; catalog number: sc-65497, Santa Cruz Biotechnology, Santa Cruz, CA, USA) was applied to the sections overnight at 4 °C. Sections were then washed with PBS and incubated with appropriate secondary antibody (m-IgG2a BP-HRP, Santa Cruz Biotechnology, Santa Cruz, CA, USA) for 1 h at room temperature. These antibodies were visualized by diaminobenzidine (DAB) and counterstained with hematoxylin. Negative control slides were treated by the same steps except for incubation with the primary antibody. Tonsil sections were used as a positive control.

### Transmission electron microscopy (TEM)

Small muscle specimens were put in a mixture of 2.5% glutaraldehyde and 2% paraformaldehyde in 0.1 mol/l sodium cacodylate buffer (pH 7.4). Subsequently, the specimens were postfixed for 1 h at 4 °C in 2% osmium tetroxide in 0.1 mol/l sodium cacodylate buffer (pH 7.4). After dehydration and clearing, tissue fragments were finally embedded in epoxy resin. Semithin sections (1 μm) were cut and stained with toluidine blue. Ultrathin sections (60–70 nm) were cut using an ultramicrotome, picked on copper grids, and stained with 2% uranyl acetate and lead citrate. The specimens were then examined by a JEM-100CXi, (Jeol, Tokyo, Japan) at the electron microscope unit of the Faculty of Science, Alexandria University, Alexandria, Egypt.

### Digital morphometric study

Slides were photographed using an Olympus® digital camera (SC100) installed on an Olympus® light microscope (CX31; Japan). The obtained photos were examined with Image J 1.52a (National institutes of health, USA) to measure the percentage of degenerated muscle fibers in H & E-stained sections, the percentage area of collagen fibers in Mallory’s trichrome stained sections, and the percentage area of caspase-3 positive fibers in immunohistochemically stained slides. Six slides from each animal of the experiment in each stain were examined. Six nonoverlapping fields were randomly chosen to estimate the statistical data.

### Statistical analysis

SPSS version 26 (SPSS Inc., Chicago, Illinois, USA) was used to analyze the biochemical and morphometric data. Data normality was tested using the Kolmogorov–Smirnov test, and they were normally distributed. Quantitative results were expressed as mean ± SD and were analyzed by one-way analysis of variance test (ANOVA) followed by Tukey post-hoc test. A* p*-value ≤ 0.05 was considered statistically significant.

## Results

### Light microscopy results

#### H & E-stained sections

**Fig. 1:**
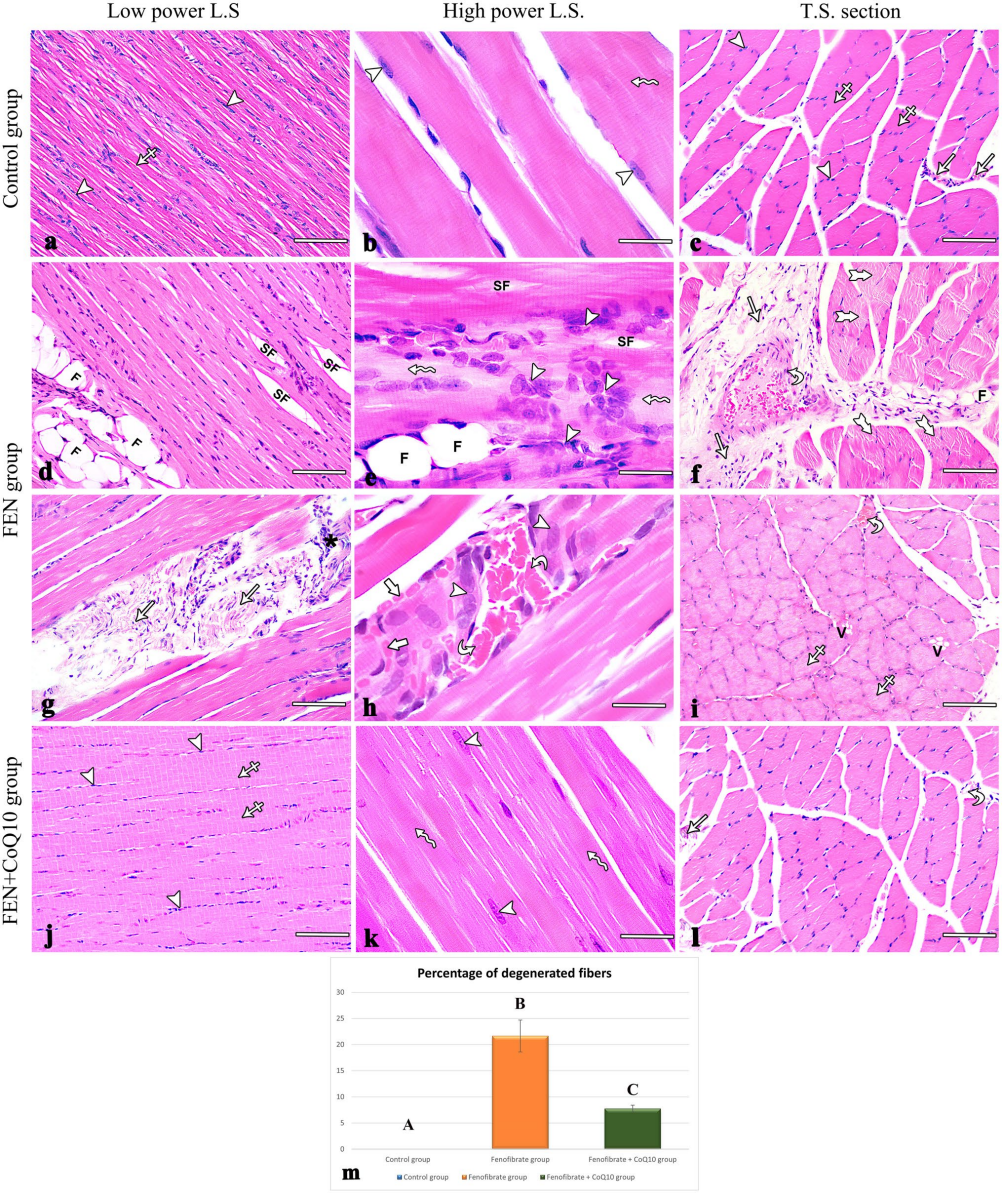
Photomicrographs of H & E-stained soleus muscle sections of all experimental rats. (**a**–**c**) the control group (**a, b**) L.S. showing parallel nonbranching muscle fibers with acidophilic cytoplasm (crossed arrow), regular transverse striations (wavy arrows), and multiple peripheral oval nuclei (arrowheads). (**c**) T.S. showing bundles of polyhedral fibers with peripheral, oval nuclei (arrowheads) and acidophilic cytoplasm (crossed arrows), separated by connective tissue perimysium (arrows). (**d**–**i**) the FEN group. (**d, e**) L.S. showing splitting of muscle fibers (SF) with fat cells (F) in between, disorganized muscle fibers with aggregated nuclei (arrowheads), and areas of lost striations (wavy arrow). (**g, h**) An excessive amount of connective tissue between the fibers (arrows) with inflammatory cellular infiltrates (black asterisk), a congested blood vessel (curved arrow), and extravasated RBCs (thick arrow). (**f, i**) T.S. showing excess connective tissue perimysium (arrows) with a congested blood vessel (curved arrow) and fat cells (F). Cytoplasm was fragmented (forked arrow), pale (crossed arrow), and vacuolated (V). (**j**–**l**) The FEN + CoQ10 group. (**j, k**) L.S. showing parallel nonbranching muscle fibers with acidophilic cytoplasm (crossed arrow), regular transverse striations (wavy arrows), and multiple peripheral oval nuclei (arrowheads). (**l**) T.S. showing bundles of muscle fibers separated by connective tissue perimysium (arrow) containing a blood vessel (curved arrow). (**m**) Statistical analysis of the percentage of degenerated fibers (mean ± SD) in the soleus muscle within the studied groups. Different letters represent a significant difference at *p*  ≤ 0.05. FEN: fenofibrate; CoQ10: coenzyme Q10; L.S.: longitudinal section; T.S.: transverse section; RBCs: red blood cells. (**a, c, d, f, g, i, j, l**) scale bar: 100 μm, (**b, e, h, k**) scale bar: 25 μm

Sections of the soleus muscle of both positive control and negative control groups had similar findings. Examination of longitudinal sections (LS) of the soleus muscle of the control group revealed the organized architecture of the muscle fibers, the structural units of the muscle. They were long, striated, and nonbranching, with elongated, multiple, peripheral vesicular nuclei and acidophilic sarcoplasm (Fig. 1a, b). Transverse sections (TS) of the muscle fibers were polygonal in shape with peripheral nuclei and acidophilic sarcoplasm. The muscle fibers were grouped into bundles surrounded by a connective tissue perimysium (Fig. 1c).

The LS sections of the FEN group revealed distorted structure of muscle fibers marked by lost striations, splitting of some muscle fibers, and condensed, centrally displaced nuclei. Inflammatory cellular infiltrates, extravasated red blood cells (RBCs), congested blood vessels and many fat cells were found between the muscle fibers (Fig. 1d, e, g, h). The TS sections of the muscle fibers contained pale, vacuolated sarcoplasm, surrounded by thickened connective tissue perimysium containing congested thickened blood vessels and some fat cells (Fig. 1f, i). The percentage of degenerated fibers was significantly higher in this group (21.66 ± 3.05; *p* < 0.001) compared with the control group (Fig. 1m).

The LS and TS sections of the FEN + CoQ10 group revealed that most of the fibers showed remarkable preservation of the organized structure compared with the FEN group (Figs. 1j, k, l). The percentage of degenerated fibers showed a highly significant decrease in this group (7.74 ± 0.66, *p*<0.001) compared with the FEN group. However, it showed a significant increase (*p* = 0.008) when compared with the control group (Fig. 1m).

### Toluidine blue-stained sections

**Fig. 2:**
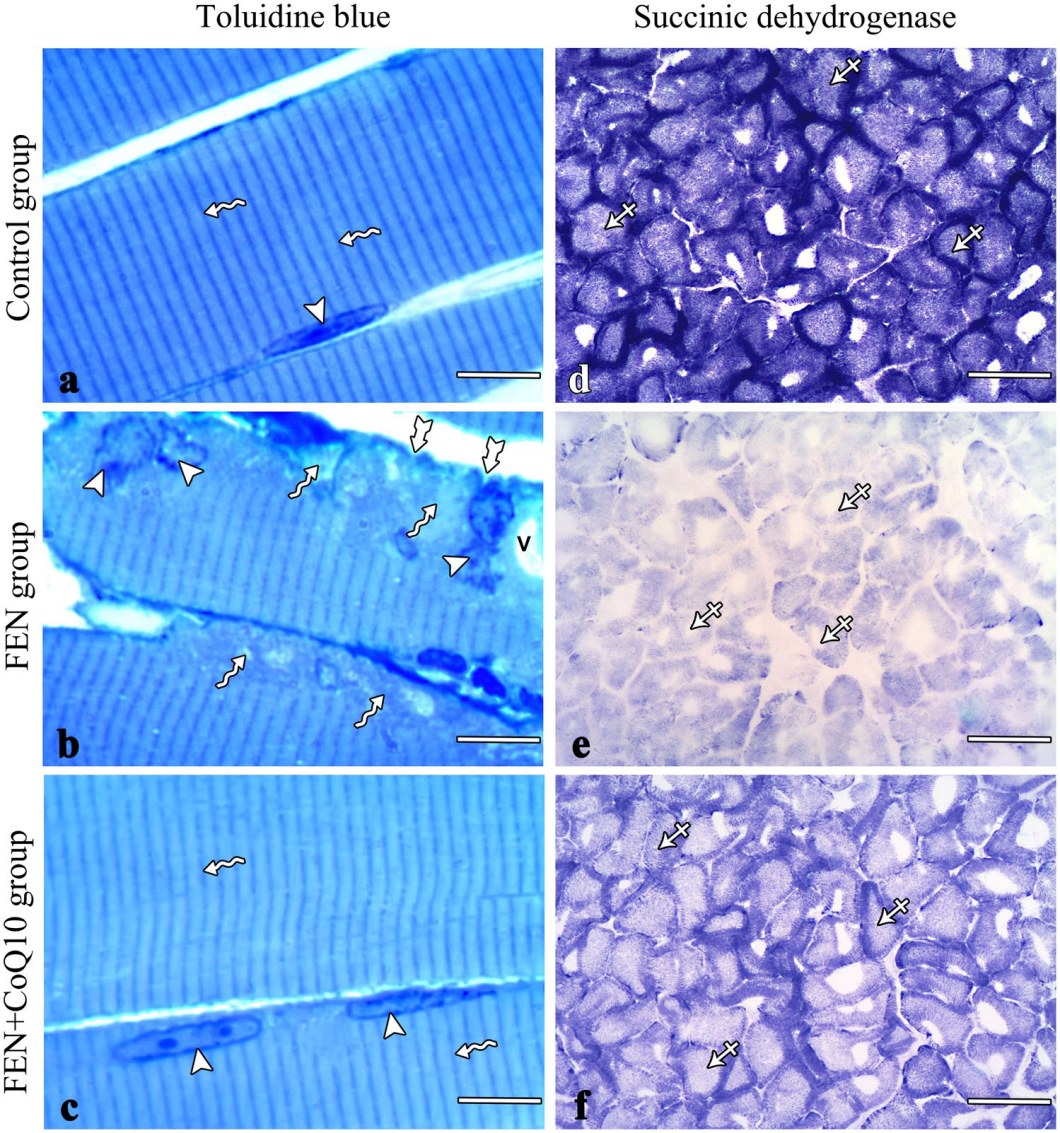
Photomicrographs of muscle sections stained with toluidine blue (**a–c**) and SDH (**d–f**). (**a**) The control semithin sections show regular striated myofibrils (wavy arrow) with elongated peripheral nuclei (arrowheads). (**b**) The FEN group shows irregular sarcolemma (bifid arrows) with irregular displaced nuclei (arrowheads), cytoplasmic vacuole (V), and areas with lost striations (wavy arrows). (**c**) The FEN + CoQ10 group shows striated myofibrils (wavy arrows) with elongated peripheral nuclei (arrowheads). (**d**) The control group shows high SDH staining intensity (crossed arrow). (**e**) The FEN group shows weak intensity (crossed arrow). (**f**) The FEN + CoQ10 group shows moderate intensity (crossed arrow). FEN: fenofibrate; CoQ10: coenzyme Q10; SDH: succinic dehydrogenase. (**a, b, c**) scale bar: 10 μm, (**d, e, f**) scale bar: 100 μm

The semithin sections of the control group stained with toluidine blue showed the regular transverse striations and elongated peripheral nuclei of the muscle fibers (Fig. 2a). The sections of the FEN group showed that the myofibrils had irregular displaced nuclei and irregular sarcolemma. The sarcoplasm contained vacuoles and areas of degeneration and lost striations (Fig. 2b). Examination of the sections of the FEN + CoQ10 group showed nearly normal muscle fibers with regular transverse striations and elongated peripheral nuclei (Fig. 2c).

### Histochemically stained sections (SDH)

In the control group, histochemical study of frozen TS of the soleus muscle for detection of SDH activity showed that this muscle consisted mainly of deeply stained fibers (type I) (Fig. 2e). Sections of the FEN group showed a weak reaction in most fibers (Fig. 2f), while those of the FEN + CoQ10 group showed a moderate increase in the intensity of the staining reaction compared with the FEN group (Fig. 2g).

### Mallory’s trichrome-stained sections

**Fig. 3:**
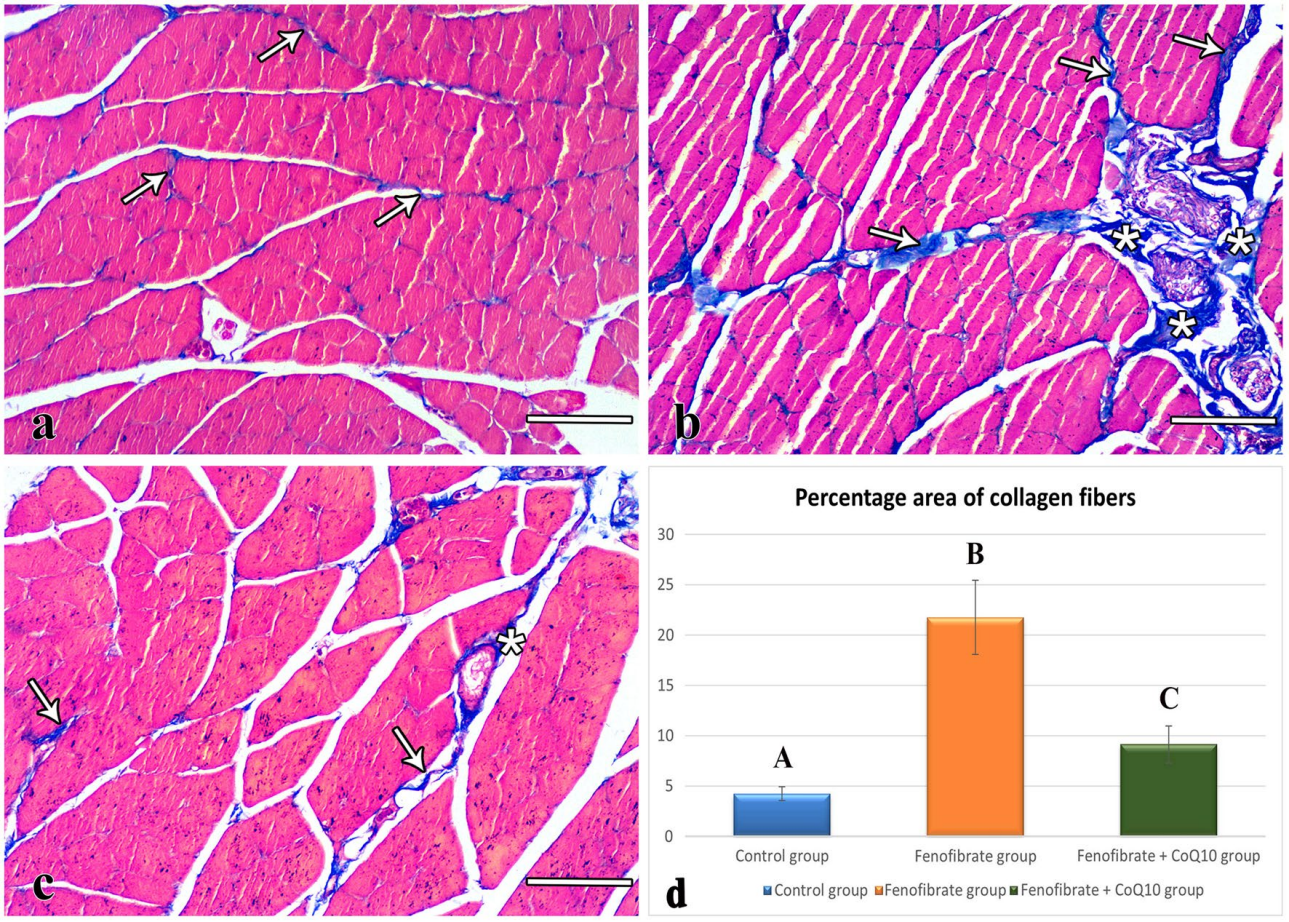
Photomicrographs of Mallory’s trichrome-stained sections. (**a**) A minimal amount of collagen fibers (arrows) between the control muscle fibers. (**b**) An excessive amount of collagen between FEN group muscle fibers (arrows) and around blood vessels (asterisk). (**c**) A little amount of collagen between the FEN + CoQ10 muscle fibers (arrows) and around blood vessels (asterisk). (**d**) Statistical analysis of the percentage area of collagen fibers (mean ± SD) within the studied groups. Different letters represent a significant difference at *p*  ≤ 0.05. FEN: fenofibrate; CoQ10: coenzyme Q10. (**a, b, c**) scale bar: 100 μm

In the control group, Mallory’s trichrome-stained TS sections of the soleus muscle showed a minimal amount of collagen fibers in between the muscle fibers and around the blood vessels (Fig. 3a). Sections of the FEN group showed an excess amount of collagen fibers in between the muscle fibers and around the blood vessels (Fig. 3b). The percentage area of collagen fibers in this group (21.75 ± 3.68, *p* < 0.001) was significantly higher compared with the control group (4.42 ± 0.98) (Fig. 3d). On the other hand, the sections of the FEN + CoQ10 group showed a little amount of collagen fibers in between the muscle fibers and around the blood vessels (Fig. 3c). The percentage area of collagen fibers in this group (9.14 ± 1.84, *p* < 0.001) showed a highly significant decrease compared with the FEN group and a significant increase (*p* = 0.042) compared with the control group (Fig. 3d).

### Immunohistochemically stained sections

**Fig. 4:**
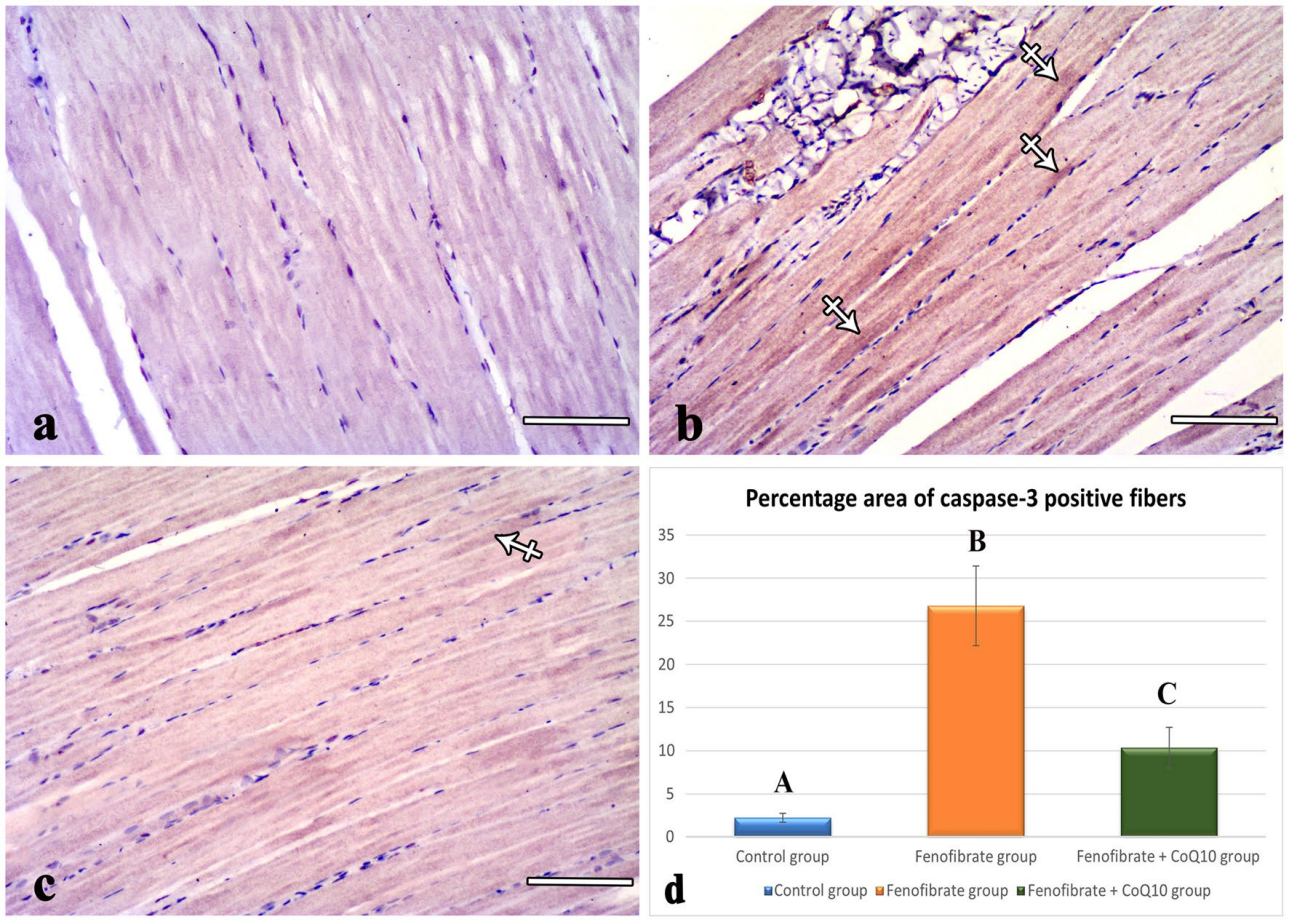
Photomicrographs of anti-caspase-3 immunostained sections. (**a**) The control muscle fibers show a negative caspase-3 immune reaction. (**b**) A positive reaction in the cytoplasm (crossed arrows) of FEN group muscle fibers. (**c**) A mild positive reaction in the cytoplasm of the FEN + CoQ10 muscle fibers (crossed arrows). (**d**) Statistical analysis of the percentage area of caspase-3-positive fibers (mean ± SD) within the studied groups. Different letters represent a significant difference at *p*  ≤ 0.05. FEN: fenofibrate; CoQ10: coenzyme Q10. (**a, b, c**) scale bar: 100 μm

Examination of anti-caspase-3-stained sections of the control group showed negative immune expression of this marker in the muscle fibers (Fig. 4a). Sections of the FEN group showed caspase-3-positive immune reactivity in the cytoplasm of some fibers (Fig. 4b). The percentage area of caspase-3 positive fibers in this group (26.79 ± 4.62, *p* < 0.001) was significantly higher compared with the control group (2.06 ± 0.29) (Fig. 4d). Sections of the FEN + CoQ10 group revealed mild positive cytoplasmic immunoreactivity (Fig. 4c). The percentage area of caspase-3-positive fibers in this group (10.35 ± 2.37, *p* < 0.001) showed a highly significant decrease compared with the FEN group; however, it showed a significant increase (*p* = 0.005) compared with the control group (Fig. 4d).

### Electron microscopy results

**Fig. 5:**
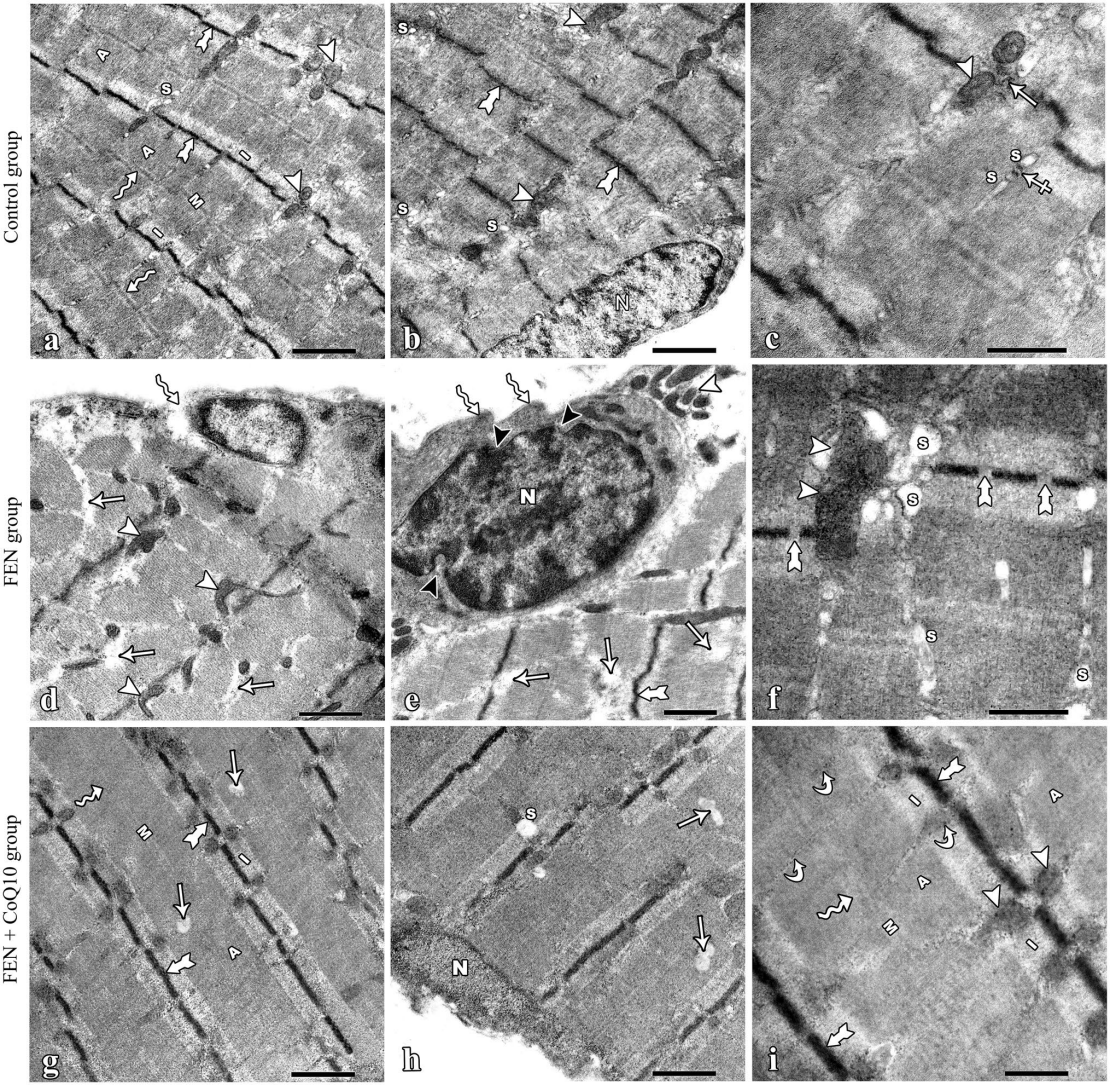
Electron photomicrographs of the soleus muscle of all experimental rats. (**a**–**c**) the control group: (**a, b**) The normal banding pattern of the myofibrils of the control rat muscle with alternating I band (I) and A band (A). Z lines (bifid arrows) appear in the middle of the I band, the H band (wavy arrow) is bisected by the M line (M). Mitochondria (arrowheads) are seen between the myofibrils. The sarcoplasmic reticulum (S) is seen at the A–I junction. The nucleus (N) is peripheral, oval, and euchromatic. (**c**) The characteristic triad of skeletal muscle formed of one T tubule (crossed arrow) and two cisternae (S) of sarcoplasmic reticulum. Also, glycogen granules (arrow) and mitochondria (arrowhead) between the myofibrils. (**d**–**f**) the FEN group: (**d**, **e**) The myofibrils show irregular and disrupted sarcolemma (wavy arrow), areas of degeneration (arrows), irregular Z lines (bifid arrow), abnormal-shaped mitochondria (arrowheads), and irregular-shaped nucleus (N) with the irregular nuclear envelope (black arrowhead). (**f**) The myofibrils show dilated sarcoplasmic reticulum (S), degenerated mitochondria (arrowheads), and disrupted Z lines (bifid arrows). (**g**–**i**) The FEN + CoQ10 group myofibrils show preserved normal banding pattern with alternating I bands (I) and A bands (A). Z lines (bifid arrows) appear in the middle of the I band and M line (M) appears in the middle of the H band (wavy arrow). Mitochondria (arrowheads) are arranged in pairs around Z lines and a few glycogen granules (curved arrows) are seen. The nucleus (N) is peripheral and oval. Notice the presence of few foci of degeneration (arrow) and dilated sarcoplasmic reticulum (S). FEN: Fenofibrate; CoQ10: coenzyme Q10. (**a, b, d, e, g, h**) scale bar: 0.1 μm. (**c, f, i**) scale bar: 500 nm

Examination of ultrathin sections of the control group soleus muscle showed normal ultrastructure appearance of myofibrils filling the sarcoplasm of the muscle fiber. They were arranged parallel to the longitudinal axis of the myofiber. They showed regularly arranged, alternating light (I) and dark (A) bands. A pale narrow region, the H band, was observed transecting the A band. The dark M line bisected the H band, whereas the Z line bisected the I band. Sarcomeres were detected between two successive Z lines. Mitochondria were seen between the myofibrils on both sides of the Z line. The sarcoplasmic reticulum was clearly seen at the A–I junction. Elongated euchromatic nuclei were seen under the sarcolemma **(**Fig. 5a, b). The characteristic triad of skeletal muscle was clearly seen, consisted of one T tubule and two cisternae of the sarcoplasmic reticulum (Fig. 5c).

Ultrathin sections of the FEN group muscle showed that myofibrils had irregular, disrupted sarcolemma. Some nuclei appeared irregular with irregular nuclear envelope. Sarcoplasm showed areas of degeneration and disruption of Z line. Some mitochondria appeared abnormal, degenerated, and grouped below the sarcolemma (Fig. 5d, e). The sarcoplasmic reticulum was dilated (Fig. 5f).

Examination of ultrathin sections of the soleus muscle of the FEN + CoQ10 group showed marked improvement in the structure of the myofibrils. Most of the myofibrils appeared nearly normal with peripheral oval nuclei and regular, alternating I and A bands. Z lines appeared in the middle of the I bands and M lines appeared in the middle of the A bands. Mitochondria were arranged in pairs around Z lines. However, some myofibrils showed few foci of degeneration and disrupted Z line. Some myofibrils had dilated sarcoplasmic reticulum (Fig. 5g, h, i).

### Biochemical results

#### Serum creatine kinase level

**Fig. 6:**
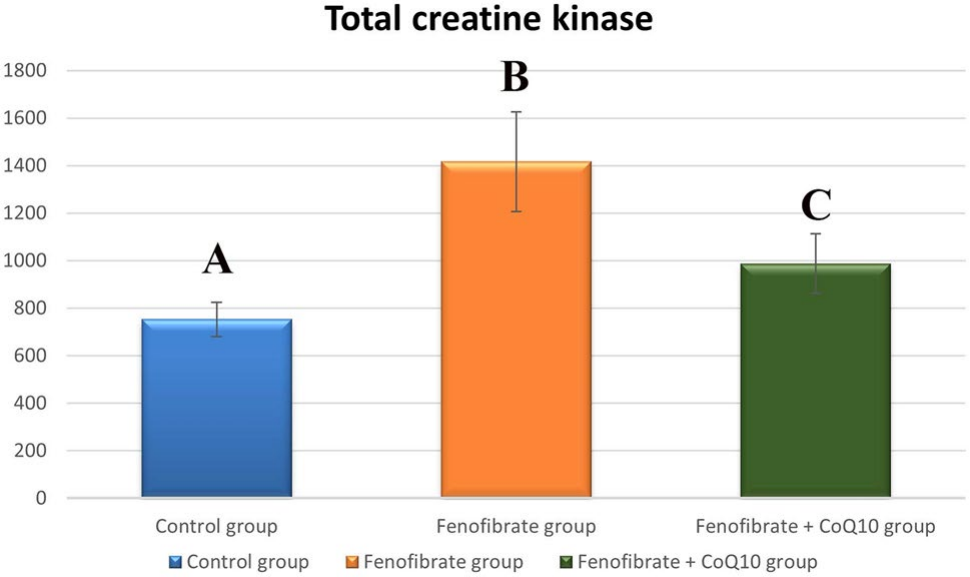
Statistical analysis of the total CK level (mean ± SD) within the studied groups. Different letters represent a significant difference at *p*  ≤ 0.05

The total CK level was significantly higher in the FEN group (1416.47 ± 210.62, *p* < 0.001) compared with the control group (753.51 ± 72.06), while it showed a highly significant decrease in the FEN + CoQ10 group (987.54 ± 125.73, *p* < 0.001) compared with the FEN group. However, it showed a significant increase in the FEN + CoQ10 group (*p* = 0.011) compared with the control group (Fig. 6).

## Discussion

Fibrates are lipid-lowering drugs that are used to control elevated levels of lipids in patients with uncontrolled hyperlipidemia. Despite their beneficial effects on lipids, fibrates have undesirable side effects on clinical outcomes, mainly affecting skeletal muscle and causing myopathy. This may range from an asymptomatic increase in plasma CK level or simple myalgia to life-threatening rhabdomyolysis. These side effects should be considered and must be recognized early and properly managed to prevent drug-related morbidity and mortality (Ajima et al. 2021) .

Fibrates are agonists of peroxisome proliferator-activated receptor alpha (PPAR-α), a nuclear transcription factor, which upregulates the synthesis of lipoprotein lipase, apolipoprotein A-I and fatty acid transport protein. It also downregulates apolipoprotein C-III, which is an inhibitor of lipoprotein lipase, resulting in a decrease of triglycerides and elevation of high-density lipoproteins (HDL) (Chhetry and Jialal 2020). In human and mice, activation of PPAR-α in skeletal muscles commonly causes muscle weakness and myalgia or muscle breakdown. This may be due to transactivation of muscle protease genes, with enhanced expression of proteases leading to myopathy. It could also be due to tissue damage and oxidative stress from an elevation of mitochondrial and peroxisomal β-oxidation. Type I muscle fibers are more sensitive to this effect (Xi et al. 2020).

In this study, administration of FEN resulted in degenerative changes of muscle fibers, represented by splitting of muscle fibers, lost striations, darkly stained, centrally displaced nuclei, and pale, vacuolated, sarcoplasm. Congested vessels, extravasated RBCs, and numerous adipocytes were also observed. This was accompanied by increased level of serum CK, which is a beneficial marker for skeletal muscle diseases. Similar findings were reported by Okada et al. (2009). It was reported that induced activity of PPAR-α by FEN may lead to increased peroxisomal β-oxidation and consequent oxidative stress injury and damage of muscle tissue (Phua et al. 2018).

Coenzyme Q10 (CoQ1) is a lipid-soluble compound that is naturally synthesized in smooth endoplasmic reticulum by the mevalonate pathway (Awad et al. 2018). Arenas-Jal et al. (2020) and Botelho et al. (2020) mentioned that CoQ10 has a fundamental function in bioenergetics of mitochondria because of its powerful antioxidant activity. It has been considered as a potential candidate for the treatment of various diseases where oxidative stress plays a significant role, such as neurodegenerative disorders, cancer, diabetes, and cardiovascular diseases. Therefore, CoQ10 was used in this study to assess its effectiveness in alleviating the structural changes that occurred after FEN administration.

Administration of CoQ10 in FEN-treated rats showed remarkable preservation of the normal structure of most muscle fibers. The improvement in this group could be due to the antioxidant properties of CoQ10. Al-Megrin et al. (2020) found that CoQ10 works as a potent lipophilic antioxidant and free radical scavenger. This could occur via two mechanisms; a direct antioxidant effect via inhibition of formation and spread of reactive oxygen species (ROS), which cause oxidative stress owing to their deleterious effects on DNA, proteins, lipids, and overall mitochondria dysfunction, and an indirect effect via increasing the synthesis of other key antioxidants such as ascorbate (vitamin C) and tocopherol (vitamin E).

Ulla et al. (2017) and Cirilli et al. (2021) stated that CoQ10 lowered the level of plasma markers of muscular damage, the CK level, which was in line with our results. This was also in agreement with previous work that reported that CoQ10 could reduce CK activity in isoprenaline-induced cardiac hypertrophy and cardiotoxicity in rats (Ghule et al. 2009).

Fat cells were seen between muscle fibers of the FEN group. This might be due to mitochondrial respiratory chain dysfunction that could be a predisposing factor for lipid deposition between the muscle fibers, causing myopathy. Previous research has shown that skeletal muscle toxicity induced by fibrate might be caused by inhibition of the mitochondrial respiratory complex I, leading to impaired mitochondrial functions (Bodie et al. 2016).

Muscle fibers in FEN-treated rats were separated by an excessive amount of connective tissue containing inflammatory cellular infiltrate. This could be due to muscle damage that triggers the release of many growth factors, such as platelet-derived growth factor and transforming growth factor-β1 (TGF-β1) that start the fibrotic process via synthesizing collagen by fibroblasts, as reported by Mehanna et al. (2019). This could also be due to oxidation compounds such as lipid peroxidation products that stimulate α-collagen expression and collagen synthesis, as mentioned by Meza et al. (2017). These products also cause the release of inflammatory cytokines, including tumor necrosis factor (TNF-α) and activated nuclear factor kappa-light-chain-enhancer of activated B cell (NFκB) that resulted in an inflammatory cascade (Mokhtari et al. 2017).

CoQ10 has antifibrotic effect, evidenced by a significant decrease in the amount of collagen fibers between the fibers. It also diminished the inflammatory cellular infiltrate. This was supported by other studies that have mentioned that the antifibrotic effect of CoQ10 could be due to altered profibrotic gene expression and ROS scavenging (Olama et al. 2018). Ekeuku et al. (2020) and Mohamed and Said (2021) found that CoQ10 possesses antiinflammatory and antioxidant properties: it lowers the level of inflammatory mediators such as TNFα, interleukin-6 (IL-6), and C-reactive protein (CRP). This occurs through inhibition of NFκB, the key regulator of inflammation. Al-Megrin et al. (2020) stated that CoQ10 also prevents the migration of leukocytes, macrophages, and monocytes, which are mainly responsible for production of proinflammatory cytokines.

The SDH activity was markedly diminished in the FEN-treated rats. FEN has a selective effect on type I muscle fibers via upregulation of genes of beta-oxidation of fatty acid. This is in line with the major clinical aim of hypolipidemic drugs, which is to promote beta-oxidation and fatty acid absorption. This upregulation results in a metabolic transition of the energy fuel from glucose to fatty acid that may be a cause of FEN-induced muscle toxicity. Previous research has found that clofibrate, another type of fibrate, could induce toxicity, selectively affecting type 1 predominant soleus muscle (Bodie et al. 2016).

The SDH activity showed marked enhancement after CoQ10 administration. This was endorsed by Mohamed et al. (2019) who stated that CoQ10 is the key cofactor in the electron transport chain via regulating the oxidant/antioxidant balance; thus, it has a protective effect on mitochondria.

In this work, enhanced apoptosis, evidenced by increased immuno-expression of caspase-3, was detected in muscle fibers in FEN-treated rats. This was in agreement with Ahmed et al. (2017) who referred this effect to increased mitochondrial membrane permeability caused by FEN-induced oxidative stress, which results in the release of cytochrome C into the cytosol. Apoptosis is triggered when cytochrome C activates caspase-9, which then cleaves and activates procaspase-3 (Soliman et al. 2017).

In the current study, CoQ10 minimizes caspase-3 immunoreactivity. This is in line with Sumi et al. (2018) and Fatima et al. (2021) who stated that CoQ10 inhibited the activation of caspase-3 and the release of cytochrome c from mitochondria via stabilizing the mitochondrial membrane, thus preventing the release of apoptotic mediators. El-Khadragy et al. (2020) determined that CoQ10 suppressed the expression of proapoptotic genes as caspase-3 and *Bax*, and stimulated the expression of the antiapoptotic gene *Bcl2*. In addition, it prevents translocation of nuclear apoptosis-inducing factors.

Ultrastructural examination of muscle fibers of FEN-treated rats proved the degenerative changes seen by light microscopy. There were areas of degeneration of myofibrils, disrupted Z lines, irregular nuclei, irregular nuclear envelope, and disrupted sarcolemma. The effect of FEN on the structure of membranes could be explained by the defect in the cholesterol-like molecules of muscle membrane, which is responsible for membrane stability and integrity. Both fibrates and statins can induce upregulation of the PPAR pathway. In addition, both drugs can inhibit the biosynthesis of cholesterol via blocking mevalonate production by 3-hydroxy-3-methylglutaryl-CoA (HMG-COA) reductase (Jacobson 2009). Large irregular mitochondria, numerous, dilated sarcoplasmic reticulum and cytoplasmic vacuoles were also detected inside myofibrils. The mitochondrial morphological alterations are mostly associated with respiratory chain defects and failure of ATP production.

Phua et al. (2018) found that fibrates increase lipoprotein lipase activity, which led to decreased cholesterol levels. This affected membrane fluidity and caused dysfunction of the Na/K pump with degeneration of the membranous organelles and irreversible cell damage. In addition, this could also change the osmolarity of the cell due to improper function of ion transport systems of the cellular membranes. This could explain cytoplasmic vacuolation and dilation of organelles.

In this study, CoQ10 administration preserved the ultrastructure and the normal banding pattern of myofibrils, with few foci of degeneration. Yousef et al. (2019) found that CoQ10 stabilized the plasma membrane and other intracellular membranes via inhibiting a peroxidation chain reaction and/or picking up ROS, thus preventing membrane phospholipids peroxidation. Abdulidha et al. (2020) stated that CoQ10 could suppress ROS generation through downregulation of nicotinamide adenine dinucleotide phosphate (NADPH) oxidase expression.

CoQ10 constitutes the only lipid-soluble antioxidant endogenously synthesized. It has been proposed that the antioxidant effect of quinones in mitochondrial membranes is mediated by α-tocopherol recycling. In contrast to other antioxidants, ubiquinol can inhibit both the initiation and propagation of lipid peroxidation. CoQ10 plays an important role in the transport of protons across lysosomal membranes to maintain the optimal pH (Varela-Lopez et al. 2016).

Among the limitations of this study was the absence of an in-depth analysis of the mechanism of the protective effect of CoQ10 and a lack of in-depth biochemical investigations, such as tissue oxidative enzymes and troponin, which needs further future study.

## Conclusions

The present paper showed that CoQ10 alleviated the toxic effect of FEN on skeletal muscle. It improved the structural changes that occurred in the muscle fibers that were evidenced biochemically, histopathologically, and ultrastructurally. This improvement might be due to downregulation of caspase-3 and inhibition of factors causing fibrosis.
